# Computational Analysis of the Energetic Stability of High-Entropy Structures of a Prototypical Lanthanide-Based Metal–Organic Framework

**DOI:** 10.1021/acs.jpcc.5c04892

**Published:** 2025-10-09

**Authors:** Surbhi K. A. Kumar, Dorina F. Sava Gallis, David S. Sholl

**Affiliations:** † School of Chemical Biomolecular Engineering, 1372 Georgia Institute of Technology, Atlanta, Georgia 30332-0100, United States; ‡ Nanoscale Sciences Department, 1105 Sandia National Laboratories, Albuquerque, New Mexico 87185-1415, United States; § University of Tennessee-Oak Ridge Innovation Institute, Oak Ridge National Laboratory, Oak Ridge, Tennessee 37831-6173, United States

## Abstract

High-entropy materials are characterized by their complex compositions, typically comprising five or more elements in near-equiatomic proportions. Applying this concept to metal ions in metal–organic frameworks (MOFs) has paved the way for exploring a new class of high-entropy MOFs. While the compositional strategy of high-entropy materials leverages configurational entropy to aid thermodynamic stability, it also poses significant analytical challenges due to the vast compositional landscape and diverse phases that these materials can adopt. We present a computational study of several complexities associated with selecting potential high-entropy versions of a prototype lanthanide-based MOF. We compute the energetics of metal mixing of these heterometallic MOFs using density functional theory (DFT) and machine learning interatomic potential (MLIP) methods. The use of MLIP methods allows a systematic exploration of the convex hull of thermodynamically stable MOF structures containing up to 5 distinct metals.

## Graphical abstract

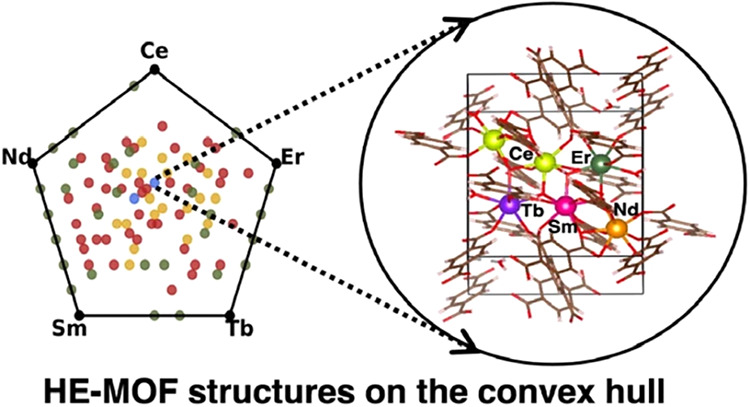

## Introduction

Metal–organic frameworks have emerged as highly adaptable materials with extremely large surface areas and adjustable pore sizes. These crystalline materials are composed of metal ions or clusters coordinated to organic linkers to form one-dimensional (1D), two-dimensional (2D), or three-dimensional (3D) structures.^1,2^ With the advancement in the design of new materials and functionalization strategies, a subclass of metal–organic frameworks (MOFs) known as mixed-metal MOFs (MM-MOFs) or heterometallic MOFs has emerged.^3–6^ This approach is intended to leverage the distinct properties of each metal to enhance the overall MOF performance. By combining different metals, MM-MOFs can exhibit a broader range of chemical and physical properties than their homometallic counterparts.^7,8^ Although most heterometallic MOFs to date involve two species of metals,^9^ mixing three or more metal ions into a single framework structure can create interesting MOFs with more complex compositions.

The inspiration for MM-MOFs and other multicomponent materials can be derived from high-entropy alloys, which were first introduced in 2004.^10,11^ Yeh et al.^10^ suggested that the presence of multiple elements can potentially increase the configurational entropy of mixing in a way that can even overcome a positive formation enthalpy, an observation that has now been extensively explored in metal alloys and nanoparticles.^12,13^ By analogy with these materials, MOFs with multiple metal species are also called “high-entropy” MOFs (HE-MOFs).^14–17^

As with metal alloys, it is not obvious that HE-MOFs can be thermodynamically stable, since they could also phase separate into many potential phases with a lower number of components at equilibrium. Although the configurational entropy of mixing can play a role in the solid solution formation, several studies have demonstrated that only maximizing the configurational entropy is inadequate.^18–20^ Therefore, it is essential to examine the relative enthalpic and entropic contributions to the overall thermodynamic stability for a high-entropy material.

Ground state density functional theory (DFT) level calculations are often used to compute the enthalpy of mixing and formation energies to predict the stability of solid materials. However, the multidimensional compositional space of potential competing phases makes computational screening for a stable HE-MOF challenging. In an example for metal alloys, Chen et al.^21^ investigated 658,008 candidates for single-phase equimolar quinary alloys by using combinations of 40 metallic elements. They identified only 30,201 (∼5% of the total candidates) as potential single-phase equimolar alloys.

The high computational cost of using DFT to search for high-entropy compositions motivates the development of other high-throughput screening methods. Recent advances in machine learning interatomic potentials (MLIPs) and related methods could offer one way to address this challenge. General-purpose MLIPs can be used as alternatives for DFT calculations in exploring the compositional space of HEMs.^22–25^ These potentials are, generally, trained on large data sets of numerous inorganic compounds. Although significant challenges remain in the generalization of these potentials, they still serve as a useful tool to explore questions that would be difficult to directly tackle with DFT. For instance, Vazquez et al.^26^ used a Graph Neural Network (GNN) model, leveraging the M3GNet model, along with a cluster expansion method to investigate short-range ordering in (Cr,Hf,Mo,Ta,Ti,Zr)­B_2_ and the refractory high-entropy alloy AlHfNbTaTiZr.

Little information is currently available about the potential formation of HE-MOFs from either experiments or simulations. Therefore, there exists a gap in our current understanding of the key factors involved in the synthesis or design of HE-MOFs. In this work, we use DFT and MLIPs to analyze the configurational landscape of HE variants of a representative, case study lanthanide (Ln)-based MOF. Our results for this specific MOF illustrate some of the physical principles that must be considered when searching for stable HE-MOFs and suggest directions for the future development of this interesting class of materials.

## Materials and Methods

### Prototype Selection

For an initial investigation, we consider a single MOF structure as a prototype to examine the range of effects that can occur with HE-MOFs. For this purpose, we focused on an Ln-MOF crystal that was synthesized experimentally in a homometallic form by Weng et al.^27^ This structure was used as a prototype MOF in previous bimetallic MOF-related studies by our group.^28,29^ The parent structure of this MOF is [Yb_6_(μ_3_–OH)_4_ (benzene dicarboxylic acid)_7_(H_2_O)_4_].2H_2_O. This structure has a hexanuclear cluster core and three different coordination environments for the metal atoms (Yb in the original structure) with a formal charge of +3. The cif file of the crystal structure (structure code: REQDIB) was obtained from CCDC.^30^ The unit cell of the MOF is shown in Figure 1. Our previous DFT calculations showed that the geometry of the DFT-optimized MOF using the PBEsol functional is in good agreement with the experimentally reported structure.^28^

**Figure 1:**
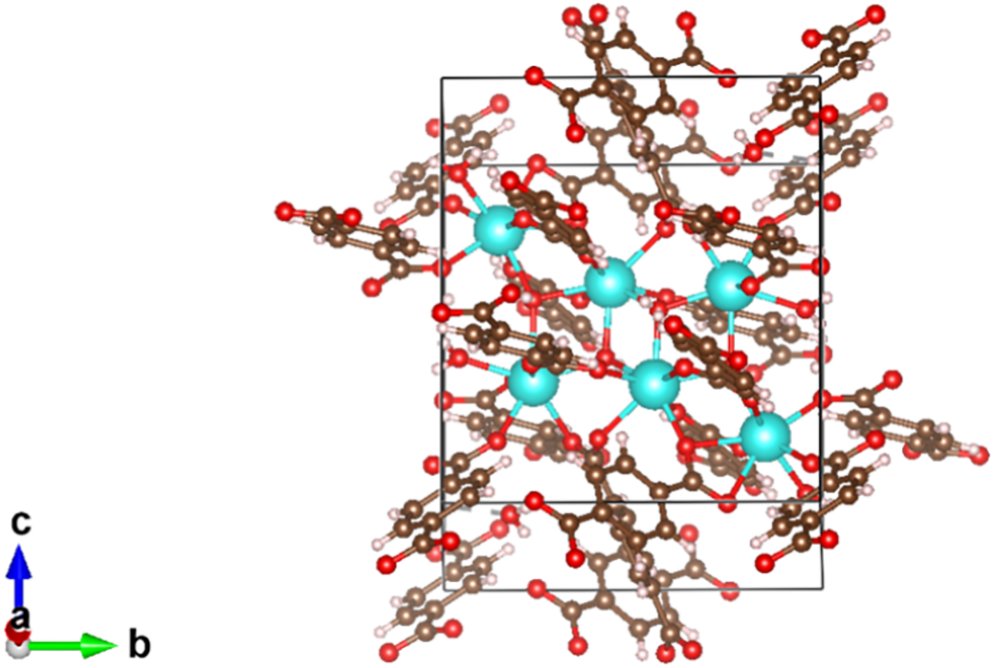
Unit cell of REQDIB, showing all of the same metal species atoms in the hexanuclear cluster and viewed along the *a* axis. Ytterbium (Yb) atoms are shown in cyan, carbon atoms are shown in brown, oxygen atoms are shown in red, and hydrogen atoms are shown in light pink.

Due to the hexanuclear metal nodes with varying coordination environments within this MOF, we can explore numerous structures of different metal compositions at the unit cell level. To screen HE derivatives of this prototype MOF, we examined the structures of this MOF with up to five different metals. Table 1 lists the number of distinct configurations that can be generated by placing metals in different permutations in a single unit cell. Even though some of these configurations are equivalent by symmetry, it is clear that thousands of distinct configurations are possible. To fully consider whether a HE phase is stable, it is necessary to consider the full range of compositions listed in Table 1 to form the overall convex hull in the composition space.^31,32^ This observation highlights the potential value of using relatively computationally efficient methods such as MLIPs instead of DFT calculations, provided that the force fields are precise enough to make useful predictions.

**Table 1:** Total Number of Possible Configurations of the Prototype MOF with up to Five Different Metals in the Unit Cell with Six Distinct Metal Positions

no. of species in the unit cell	metal combinations	composition ratios	total structures
2	10	3:3,1:5,2:4	620
3	10	1:3:2,2:2:2,1:1:4	5400
4	5	1:1:1:3,1:1:2:2	7800
5	1	1:1:1:1:2	1800

### Density Functional Theory (DFT) Calculations

Density functional theory is a widely used quantum-mechanical technique and has been extensively applied to MOFs.^33^ We used DFT as implemented in Vienna Ab initio Simulation Package (VASP)^34,35^ v5.4.4 using projector-augmented wave (PAW) potentials^36,37^ to perform spin-restricted ground state electronic structure calculations as well as geometry optimizations of crystalline MOF structures. We used Ln_3 pseudopotentials (potpaw_PBE.54) for all of the lanthanides (except La), along with large core pseudopotentials for all of the other elements with an energy cutoff of 520 eV. Both energy and force convergence criteria were used with an accuracy of 10^–5^ eV and 0.01 eV/Å, respectively. Since each of the cell dimensions in all of the lattices was more than 10 Å, reciprocal space was sampled only at the Γ-point. A Gaussian smearing with a width of 0.01 eV was used. Nazarian et al.^38^ have previously shown that the structural properties of MOFs are similar for a range of DFT functionals. In our calculations, the generalized gradient approximation exchange correlation functional of Perdew–Burke–Ernzerhof for solids and surfaces (PBEsol)^39^ was used because of its previously reported accuracy in predicting the physical properties of Ln-MOFs.^40–42^ Dispersion corrections were included in the calculations by using the D3 method of Grimme^43^ with Becke–Johnson damping.^44^ Structural relaxations were performed by using a conjugate gradient (CG) algorithm. For fixed lattice relaxations, only atomic positions were optimized. For a flexible lattice relaxation, we allowed the lattice constants to change along with the atomic positions in three steps: (1) a fixed lattice relaxation, (2) optimization of atomic positions, cell shape, and cell volume, and (3) reoptimization of atomic positions with fixed lattice parameters.^28,42^ Once an optimized structure was obtained, we performed a final single-point energy (SPE) calculation to obtain the total energy of the structure.

### Machine Learning Interatomic Potentials (MLIPs)

We used two general-purpose ML potentials for performing structural minimization and predicting the total energy of multimetallic MOFs. The first ML-FF we used is M3GNet v0.2.4 developed by Chen et al.^22^ based on graph neural networks with three-body interactions. M3GNet was trained on the DFT-optimized crystal structures of around 63,000 compounds encompassing 89 elements across the periodic table, from the Materials Project^45^ data set MPF.2021.2.8. In this data set, a series of two structural relaxation calculations with the Perdew–Burke–Ernzerhof (PBE) generalized gradient approximation (GGA) functional^46^ or the GGA + U method^47^ were conducted for each crystal structure. The M3GNet FF has an average mean absolute error (MAE) of 0.035 eV/atom, 0.072 eV/Å, and 0.41 GPa for the energy, force, and stress for this training set.

We also performed calculations with the Crystal Hamiltonian Graph neural Network (CHGNet) v0.3.0, a charge-informed universal potential developed by Deng et al.^25^ CHGNet was trained on the energies, forces, stresses, and magnetic moments obtained from DFT structure relaxation calculation (PBE-GGA or PBE-GGA+*U*) of around 146,000 materials forming the Materials Project Trajectory MPtrj data set.^48^ To maintain consistency of energies within the data set, a GGA/GGA + U mixing compatibility correction was applied. The MAEs for CHGNet for this training set are 0.03 eV/atom, 0.077 eV/Å, and 0.384 GPa and 0.032 μB for energy, force, stress, and magnetic moment, respectively.

The data sets used for training these models are primarily sourced from the Inorganic Crystal Structural Database (ICSD)^49,50^ and are thus strongly skewed toward inorganic materials. Neither M3GNet nor CHGNet is expected to accurately capture the effects of dispersion interactions because the conventional DFT functionals used to generate the training data do not account for these interactions. However, it is likely that these interactions are important in studying some properties of MOFs as they play a role in structural flexibility and other properties.^51^

### Energy of Mixing

The equilibrium stability of a mixed-metal phase of a MOF relative to its homometallic counterparts can be quantified by the energy of mixing^28,52^

1 $$E_{\mathrm{mix}}=E_{\mathrm{heterometallic}}-\sum_{i=1}^{N}x_{i}E_{i}$$

where *x*_*i*_ is the mole fraction of the *i*th metal in the heterometallic MOF and *E*_*i*_ is the energy of the homometallic MOF, where all of the metal nodes are occupied by the *i*th metal, and *E*_heterometallic_ is the energy of the heterometallic MOF.

With this definition, a negative mixing energy, *E*_mix_ < 0, indicates that the heterometallic MOF is energetically stable with respect to demixing into the constituent homometallic MOFs. Unless stated otherwise, energies of mixing reported below are normalized to give the energy per unit cell. In general, we calculated energies of mixing with DFT or force field total energies, giving the enthalpic contribution to the more general free energy of mixing. These energies did not include contributions to the free energy from vibrational degrees of freedom (e.g., zero-point energies). We also discuss below the inclusion of ideal configurational entropy in the free energy of mixing. Because the energy of mixing is a relative energy, it might be expected that systematic errors associated with the underlying energy calculations (e.g., the absence of dispersion interactions in the training data for an MLIP) could be subject to cancellation of errors.

A major limitation of this approach is that it cannot account for the possibly complex kinetic pathways that may control the crystallization of MOFs. This information is still useful in searching for possible HE-MOFs however, since it would likely be unwise to expend significant efforts to synthesize nominally HE-MOFs if the high-entropy forms can at best be metastable at equilibrium and therefore must be kinetically trapped during crystallization.^53,54^

## Results and Discussion

### Lanthanides and MLIP Selection

To explore the high-entropy configurational space of the prototype MOF, we selected five different metal species from the lanthanide series. We first generated homometallic derivative structures of the prototype by spanning across the series and replacing all of the Yb metal positions in the unit cell with one lanthanide at a time and performed flexible lattice relaxation (atomic positions + volume) using DFT as well as MLIPs (M3GNet/CHGNet). For the MLIPs, we used the Broyden-Fletcher-Goldfarb-Shanno (BFGS) algorithm implemented in the ASE package^55^ for relaxation. We used a conjugate gradient (CG) algorithm for the DFT approach. Similar force (0.01 eV/Å) convergence criteria were used for both MLIPs and DFT to maintain consistency. Below, we refer to MOF structures using just the total metal composition in the six core positions of the MOF to represent the entire structure for simplicity, even though each MOF structure has the chemical complexity shown in Figure 1. For example, a MOF structure with equiatomic Nd and Tb in the hexanuclear cluster is denoted as Nd_3_Tb_3_.

Direct comparisons of absolute relaxed energy values across different methods are not meaningful due to the arbitrary nature of the reference energy points used in these calculations. We did, however, examine the relative energy trends within a single computational framework for all of the single-metal MOFs. As shown in Table S1, the relative energies were calculated by taking the total energy of Tb_6_-MOF as the reference point. While the DFT relative energies were up to ±2 eV/unit cell, we observed huge outliers with the MLIPs for some of the lanthanides, particularly Eu, Gd, and Yb. This appears to be associated with the difference in the pseudopotentials used for our calculations and the training data from the Materials Project used in developing the MLIPs we tested.^45^ We used +3 valence state pseudopotentials for all of the metals in the MOF based on the coordination environment. However, the DFT calculations underlying the MLIPs used other pseudopotentials for some metals to gain better agreement with their reference thermodynamic data. To avoid possible complications associated with these outliers, we chose five lanthanides from the remaining compatible metals spanning the series, namely, Ce, Nd, Sm, Tb, and Er. Below, we also report some calculations including Pr, which is another compatible choice for the MLIPs.

After we established the metals to be used, we tested the effectiveness of MLIPs for calculating the *E*_mix_ values of the heterometallic crystalline structures of the prototype MOF. For this purpose, we generated one structure with each possible metal combination from every row of Table 1, giving us a total of 26 such structures. For a given metal combination, we arbitrarily chose a compositional ratio and a structural arrangement of the metals in the unit cell. We then performed full structure relaxation with both DFT and the MLIPs. The *E*_mix_ values obtained from the three methods are compared in Figure 2. In these calculations, the DFT energies of mixing vary from −20 to 35 kJ mol^–1^·unit cell^–1^. This energy range is similar to the previous DFT calculations for bimetallic versions of this MOF containing Yb and Nd.^28,29^

**Figure 2:**
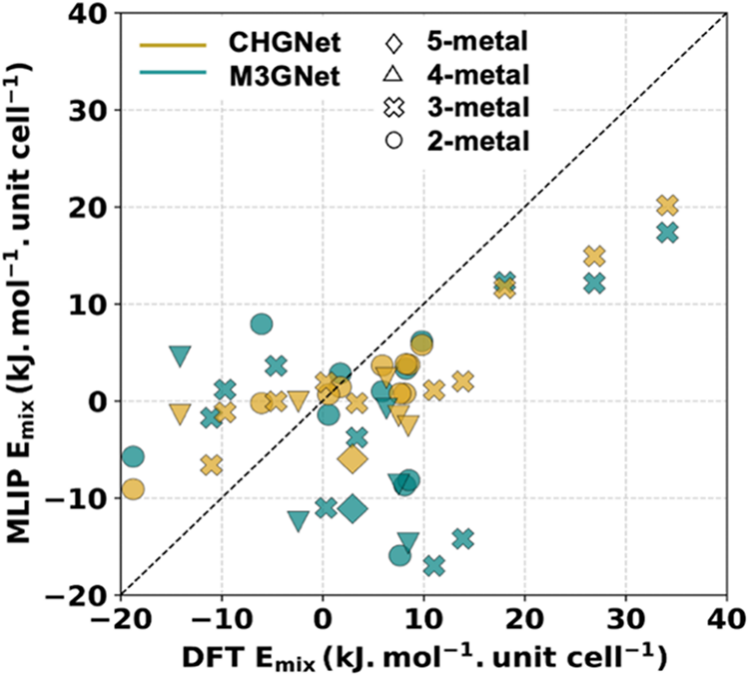
Parity plot of *E*_mix_ for MLIPs compared to DFT for 26 different configurations of the prototype MOF. The MLIP used is indicated by color, while the symbols indicate the number of metal species in the MOF’s unit cell.

Neither of the two ML potentials is fully accurate in predicting the DFT *E*_mix_ values. The mean absolute error (MAE) for the configurations we tested is 12.7 kJ mol^–1^·unit cell^–1^ (0.13 eV/unit cell) and 6.6 kJ mol^–1^·unit cell^–1^ (0.068 eV/unit cell) for M3GNet and CHGNet, respectively. Each unit cell of the MOF we used contains 144 atoms, so these MAEs are considerably smaller than the MAEs mentioned above for each MLIP relative to their full training set. This outcome is expected because *E*_mix_ is a relative energy in which it might be expected that any systematic errors associated with the MLIP would partially cancel between the configurations being compared. Based on these results, we selected CHGNet for the further calculations described below, although it is perhaps best to view results with this force field as giving semiquantitative information that is useful for exploring the large available configuration space.

We also evaluated the MAE of CHGNet-predicted *E*_mix_ values after including dispersion corrections. These calculations were carried out by combining the torch-dftd^56^ implementation of the D3 method of Grimme^43^ with Becke–Johnson damping^44^ to CHGNet. Our results showed that the MAE calculated across all 26 structures using this approach was 10.1 kJ mol^–1^·unit cell^–1^ (0.1 eV/unit cell). The inclusion of dispersion did not improve prediction accuracy, suggesting that these errors are not dominated by dispersion interactions. Therefore, we performed a subsequent analysis without including these dispersion effects.

### Convex Hull Analysis

We used the CHGNet force field to perform full structural relaxation and calculated *E*_mix_ for all of the 15,620 single unit cell structures defined in Table 1. The force field *E*_mix_ values for the relaxed MOF structures range from −15 to 25 kJ mol^–1^·unit cell^–1^, as shown in Figure 3. Using this data, we conducted a convex hull analysis on the overall 5-metal Ce–Nd–Sm–Er−Tb MOFs to identify potential stable structures. A similar approach was applied by Yang et al.^29^ to investigate the stability of bimetallic MOFs.

**Figure 3:**
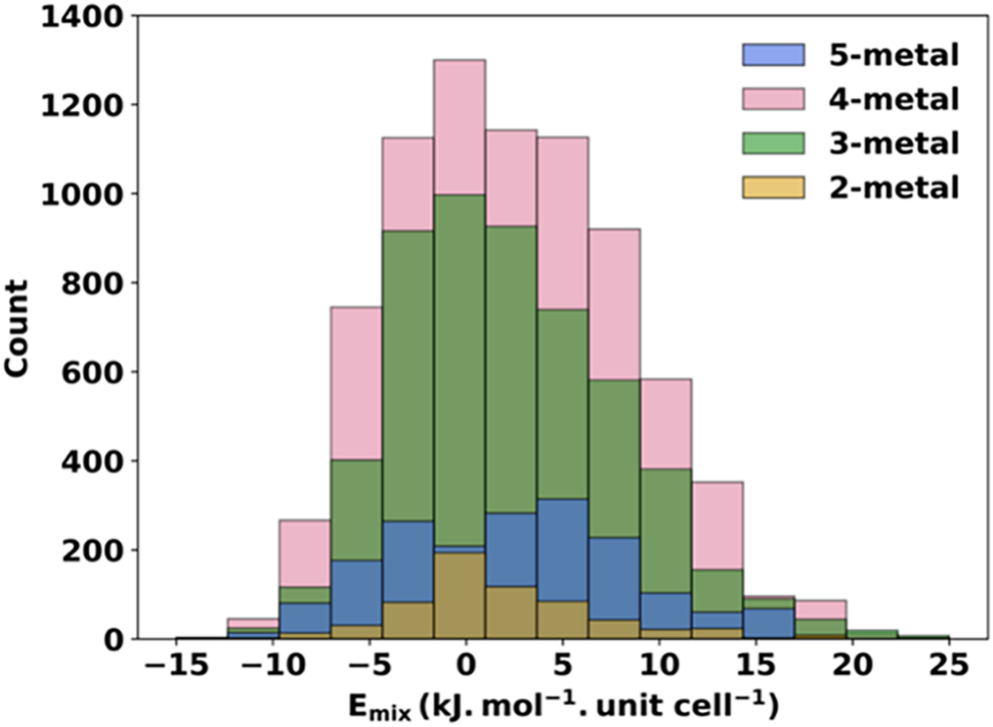
Histograms of CHGNet *E*_mix_ values for all of the single unit cell structures of the prototype MOF.

We used the PhaseDiagram class in Python Materials Genomics (pymatgen) library^57,58^ v2024.8.9 to construct the convex hull based on the CHGNet *E*_mix_ values. 97 MOFs with unique metal compositions and structures among the 15,620 we considered formed the convex hull. These compositions were projected onto a 2D regular pentagon using weighted averages of the vertex position in Figure 4a. Each vertex of the pentagon corresponds to the homometallic prototype MOF with the specified metal. 2-metal MOF compositions appear along the edges connecting adjacent metal vertices or within the pentagon for nonadjacent metals. Similarly, 3-metal, 4-metal, and 5-metal compositions are distributed within the pentagon. Of the 92 heterometallic structures on the convex hull, 29 are 2-metal, 42 are 3-metal, and 19 are 4-metal unit cell MOF structures. Only 2 of the 1800 5-metal unit cell structures included in our relaxation calculations lie on the convex hull. More details on the stoichiometry and energy of mixing of the 97 MOF structures on this convex hull are listed in Table S2.

**Figure 4:**
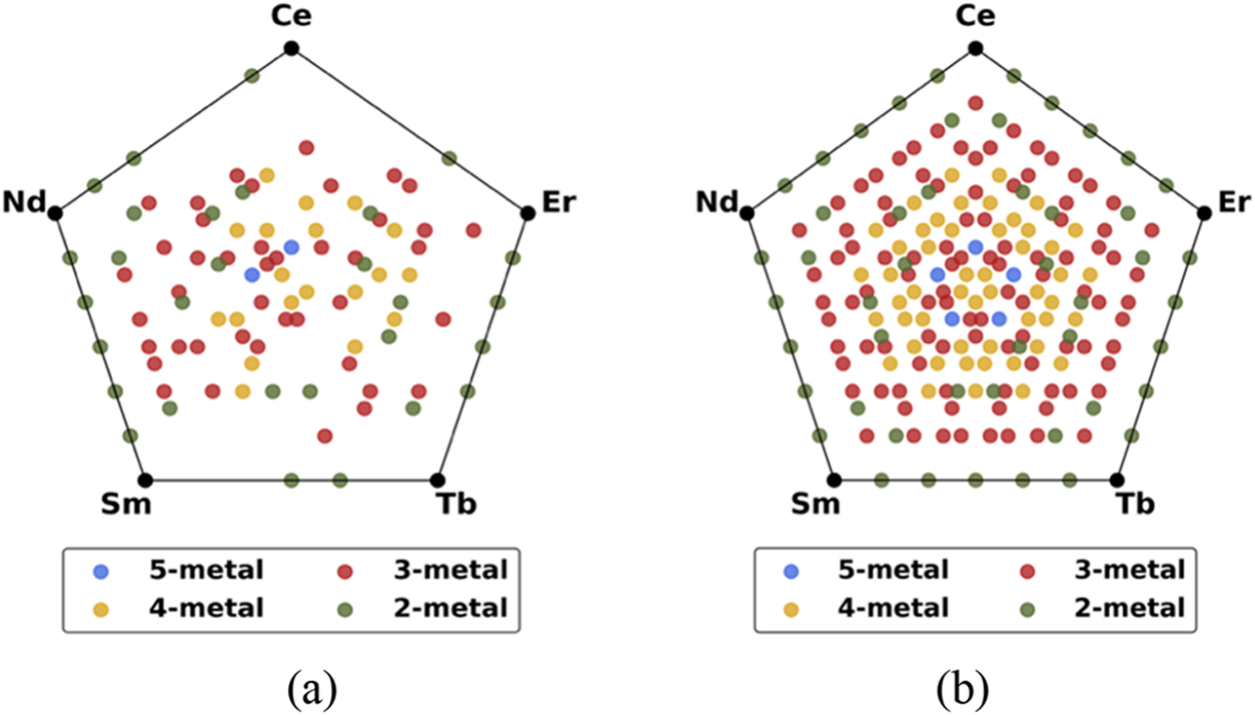
2D regular pentagon projection of Ce–Nd–Sm–Tb-Er composition space highlighting the composition of the MOF structures lying on the convex hull determined using energies from CHGNet: (a) *E*_mix_ without inclusion of configurational entropy, and (b) *E*_mix_ with ideal configurational entropy at 300 K.

We further subdivided the 92 heterometallic compositions based on whether the metal species occur in approximately equal proportions, as might be expected in a traditional HE material, or in unequal ratios. For 2-metal and 3-metal MOFs, composition ratios such as 3:3 and 2:2:2 were considered as equal ratio structures. For 4-metal and 5-metal MOFs, we considered ratios where the maximum difference in site occupancy among the metal species was ≤1 to represent approximately equal proportions. All other composition ratios were treated as unequal metal ratios. The resulting distribution is shown in Figure 5a. While most stable structures contain only 2 or 3-metal species in the MOF’s unit cell, the majority of them have metals present in unequal proportions. This correlates to the higher number of permutations possible in the composition space for unequal ratios as compared to equal ratios when considering a maximum of 3-metal species in the unit cell. However, this trend changes as we move to 4-metal unit cell MOF structures, where more permutations are possible for a near equal composition ratio of 2:2:1:1 as compared to 3:1:1:1. These observations hint that predetermining stable compositions just based on the ratios of the metal species for the formation of HE-MOFs may not be straightforward.

**Figure 5:**
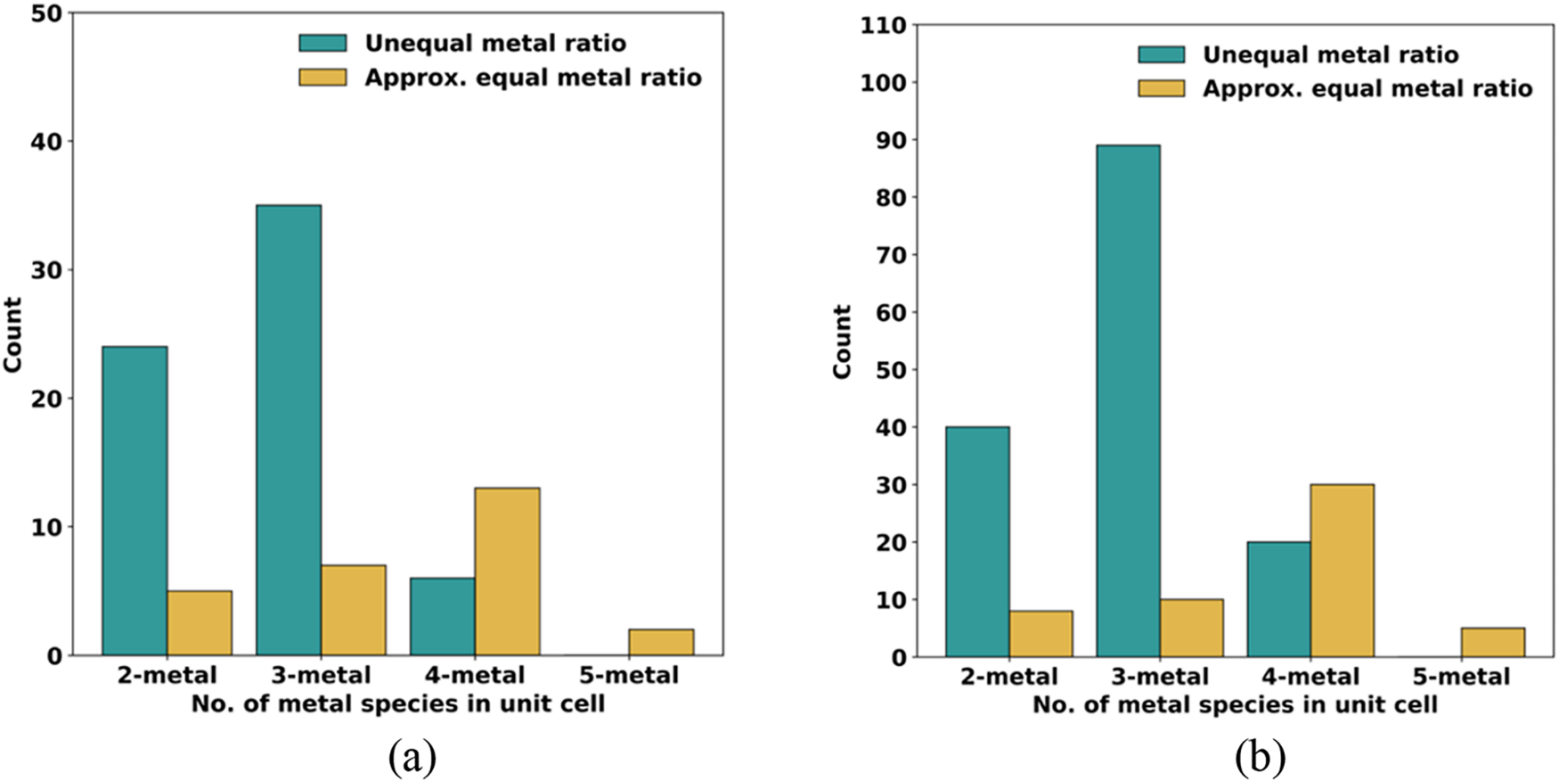
Distribution of unique stable MOF single unit cell structures lying on the Ce–Nd–Sm–Tb-Er convex hull determined using energies from CHGNet: (a) *E*_mix_ without inclusion of configurational entropy and (b) *E*_mix_ with ideal configurational entropy at 300 K.

The *E*_mix_ values used above include only enthalpic contributions. To probe the role of configurational entropy, we performed a similar analysis that added an ideal configurational entropy term,–*nR*(*x*_*i*_ × ln *x*_*i*_), where *n* is the number of different metal species and *x*_*i*_ is the mole fraction of the *i*th metal, to the *E*_mix_ values. The compositions lying on the convex hull and their distribution of metal ratios assuming a temperature of 300 K are shown in Figures 4b and 5b, respectively. Including the ideal configurational entropy increases the number of stable structures from 97 to 207. Of the 92 heterometallic structures mentioned above, 78 are also on the convex hull when including configurational entropy at 300 K. Unsurprisingly, including configurational entropy slightly favors and increases the presence of compositions with 4 or 5 metals in the unit cell on the convex hull. When this effect is included, nearly all possible compositions ranging from 2-metal to 5-metal have a unique structure lying on the convex hull. However, 49 (∼0.6%) of 7800 possible 4-metal structures and 5 (∼0.3%) of the 1800 possible 5-metal structures comprised of a single unit cell of the MOF on the convex hull still represent a very small fraction of the entire structural database. We can conclude that a randomly selected composition is not likely to be on the convex hull and would therefore be metastable.

We further checked the impact of configurational entropy at *T* = 900 K. This is an unphysically high temperature well beyond the range in which the MOF would decompose,^27^ so it gives an upper limit on this entropic effect. The temperature effects are not very significant, with the overall number of potential stable structures and their distribution being nearly the same as at 300 K, as shown in Figures S1 and S2.

Considering that a significant proportion of our unit cell structural database consists of metastable MOF structures, we determined the quantitative trends for their metastability by calculating their energy above the convex hull (*E*_hull_). For a given MOF structure with a specific composition, it denotes the vertical distance between the structure’s *E*_mix_ value and the hull energy at that composition, i.e., the minimum energy distance to the convex hull surface.^59^ *E*_hull_ is zero for a stable structure lying on the convex hull, and it is greater than zero for metastable structures.

Figure 6 shows the *E*_hull_ values for each MOF category, depending on the presence of the number of metal species. Across all cases, the *E*_hull_ values are below 35 kJ mol^–1^·unit cell^–1^ (0.36 eV/unit cell). Most 2- and 3-metal MOFs exhibit *E*_hull_ values skewed toward the lower end of the range, indicating relative proximity to the convex hull. In contrast, 4- and 5-metal MOFs have a greater proportion of relatively higher *E*_hull_ values, suggesting that these structures may readily decompose at equilibrium into more stable subclusters with fewer metal species. We repeated this analysis by incorporating ideal configurational entropy contributions (see Figure S3) and found minimal changes in the overall distribution.

**Figure 6:**
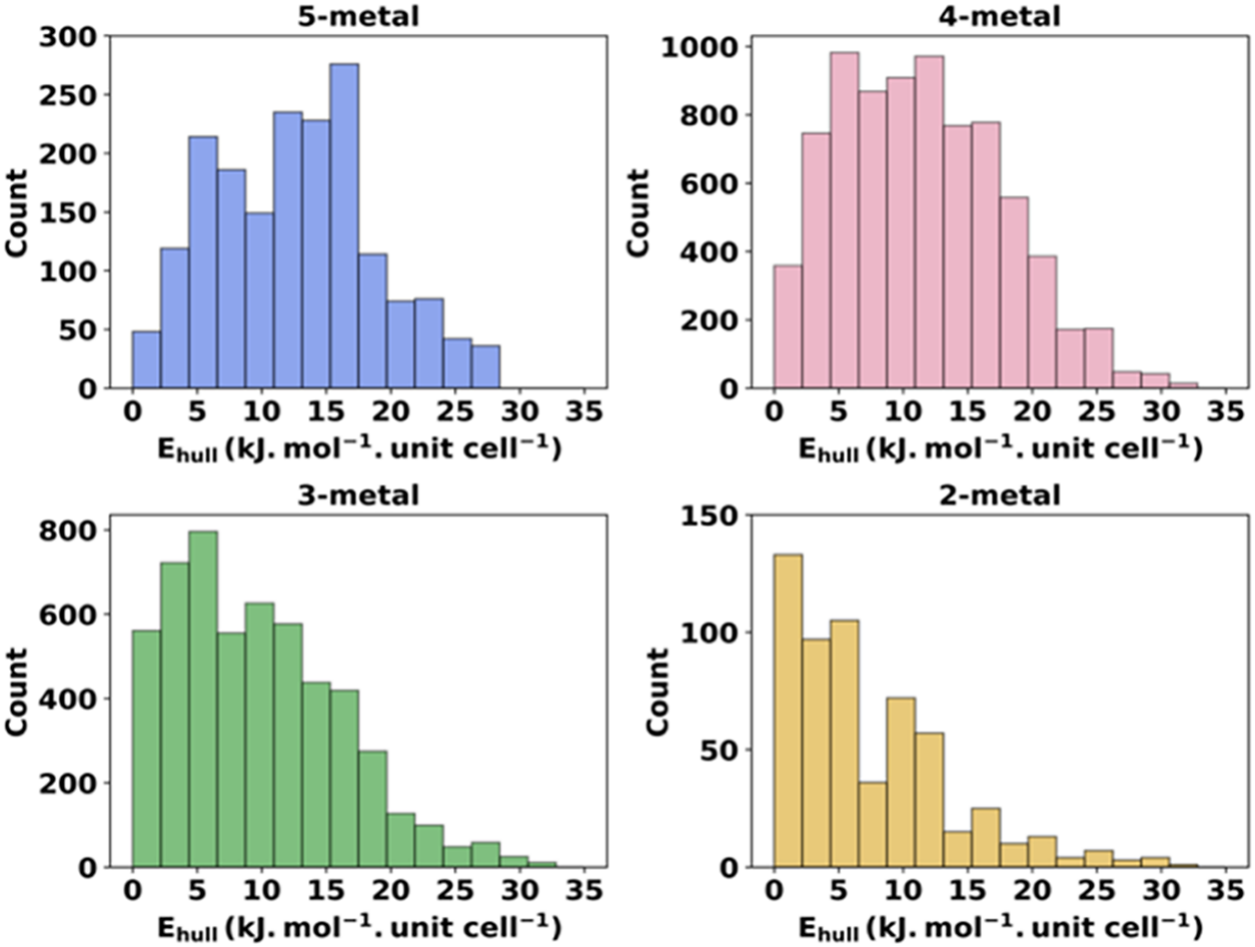
Histograms of *E*_hull_ values for Ce–Nd–Sm–Tb-Er MOFs determined for all of the metastable structures obtained from the convex hull analysis using CHGNet *E*_mix_ without inclusion of configurational entropy.

We assessed the quantitative reliability of our convex hull calculations by evaluating the DFT *E*_mix_ for a subset of the stable structures predicted by the CHGNet-derived convex hull. Since these structures cover only negative *E*_mix_ values, we also randomly selected a subset of structures with positive CHGNet *E*_mix_ values to compare them to their DFT counterparts. We divided the range of CHGNet *E*_mix_ values into three categories: −15 to −5 kJ mol^–1^·unit cell^–1^ (category 1), −5 to 10 kJ mol^–1^·unit cell^–1^ (category 2), and 10 to 25 kJ mol^–1^·unit cell^–1^ (category 3). We performed DFT calculations for eight structures in each category, with a total of 11 structures with *E*_mix_ < 0 and lying on the convex hull. The parity plot of these 24 structures are shown in Figure 7. For the structures lying on the convex hull, the *E*_mix_ values demonstrate semiquantitative agreement with their DFT counterparts, with the reported MAE of 5 kJ mol^–1^·unit cell^–1^ and are comparable to the previously reported MAE. In category 3, however, the variability in the DFT *E*_mix_ values relative to the force field predictions increases, with the MAE of 18.5 kJ mol^–1^·unit cell^–1^. After the data for these 24 structures are combined with the 26 structures in the previous section, the total MAE is 8.3 kJ mol^–1^·unit cell^–1^.

**Figure 7:**
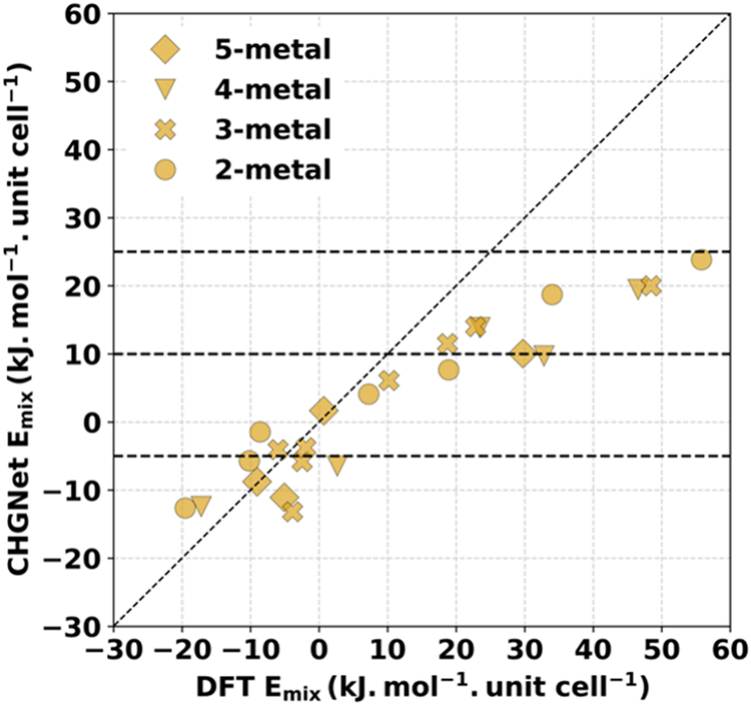
Parity plot of CHGNet and DFT *E*_mix_ for 24 heterometallic configurations of the prototype MOF within Ce–Nd–Sm–Tb-Er composition space. Eleven of the structures with CHGNet *E*_mix_ < 0 lie on the convex hull determined with this force field.

Using the DFT calculations, we also checked the bandgap for some of these stable structures from the convex hull analysis. We computed the bandgap for one representative structure each from the 5-metal, 4-metal, 3-metal, and 2-metal stable MOFs. For all of these structures, we observed similar bandgaps in the range of 2.77–2.81 eV, indicating that the metal variations do not significantly alter the bandgap characteristics in these MOFs.

#### Influence of Single-Metal Substitution

To probe the sensitivity of our convex hull analysis and related trends on the choice of lanthanides, we substituted Ce with Pr and performed an additional set of convex hull calculations on the Pr–Nd–Tb–Sm–Er MOFs. Specifically, we recreated the parity plot presented in Figure 2 by substituting Pr-based structures in place of Ce across all compositions and subjected them to structural relaxation using both CHGNet and DFT. The resulting MAE for the overall 26 structures is 5.0 kJ mol^–1^·unit cell^–1^ (0.052 eV/unit cell) and the related parity plot is shown in Figure S4.

Furthermore, we evaluated the CHGNet *E*_mix_ values for all of the unit cell structures for the Pr–Nd–Sm–Tb–Er MOFs. Figure S5 shows the histogram of these *E*_mix_ values ranging from −15 to 25 kJ mol^–1^·unit cell^–1^. We performed a comprehensive convex hull analysis and found that a total of 113 MOF compositions and structures lie on the convex hull (see Table S3), with no 5-metal compositions. After inclusion of ideal configurational entropy at 300 K, the number increased to 204 with five 5-metal MOF structures. The compositions lying on the convex hull and their distribution of metal ratios with the additional effect of ideal configurational entropy are shown in Figures S6 and S7. The histograms showing the distribution of *E*_hull_ values for the metastable structures are reported in Figure S8. Finally, we compared CHGNet and DFT values for the *E*_mix_ parity plot for a subset of the stable structures lying on the CHGNet-derived convex hull, as shown in Figure S9. The MAE for structures lying on the convex hull is 2.6 kJ mol^–1^·unit cell^–1^, while the overall MAE for the 24 structures is 6.7 kJ mol^–1^·unit cell^–1^. After combining the data for these 24 structures with the 26 structures in Figure S4, the total MAE is 5.9 kJ mol^–1^·unit cell^–1^.

Even though there are minor quantitative differences, the general trends in the convex hull analysis for Pr–Nd–Sm–Tb-Er HE−MOFs are consistent with those observed for Ce–Nd–Sm–Tb−Er HE–MOFs, reinforcing the transferability of these conclusions across other lanthanides with comparable relative energies. We observed lower MAE values after substituting Ce with Pr in our prototype MOF structures, consistent with the results in Table S1.

### Impact of Metal Short-Range Ordering

Even with identical compositions, different configurational arrangements of the metal species can lead to notable differences in *E*_mix_. For example, MOFs where the metals in the unit cell have composition CeNd_2_SmTbEr had *E*_mix_ varying from −16 to +16 kJ mol^–1^·unit cell^–1^. It can be useful to examine this situation in terms of short-range order (SRO) that arises due to the tendency of atoms of similar species to cluster together. SRO has been shown to influence the phase stability in multicomponent HEMs.^60–62^

It is challenging to capture SRO phenomena at the unit cell scale in the MOF we studied due to the limited number of atomic positions available in the computational volume. These effects can be explored, however, by considering larger supercells. This complexity has been previously reported in a study involving a 4 × 4 × 4 supercell of a bimetallic Ln-MOF M-TCPB.^29^ It is possible that these effects are further pronounced in HE-MOFs due to the presence of multiple metal species. We performed a series of calculations to probe these effects in the prototype HE-MOF that we considered.

To investigate the variation in mixing energy associated with compositional variation beyond the unit cell level, we algorithmically explored the properties of supercells with more than one unit cell. These calculations were performed as follows:

1. We take a structurally relaxed unit cell and generate a supercell containing multiple unit cells.
2. At each iteration, we generate new structures by swapping the positions of two metals of different species. We perform this single position swap for all possible metal combinations, generating a set of new structures.
3. We perform structural relaxation for all of these new structures and calculate their *E*_mix_ values. For simplicity, we only relaxed the atomic positions while maintaining constant volume in these calculations.
4. After each iteration, we determine the lowest *E*_mix_ value at that step, and the corresponding structure is used for the next iteration. In the next iteration, we follow the same approach to further create new configurations.

To demonstrate this approach, we selected a structure with the composition CeNd_2_SmTbEr and an initial *E*_mix_ = −1.3 kJ mol^–1^·unit cell^–1^. This unit cell structure lies above the convex hull and is metastable with *E*_hull_ = 9.7 kJ mol^–1^·unit cell^–1^. We generated its 2 × 1 × 1 supercell and performed 10 iterations of the above approach. The initial and final configurations are shown in Figure 8. After multiple metal position swaps, the final configuration has a significantly lower final *E*_mix_ = −7.4 kJ mol^–1^·unit cell^–1^. The two hexanuclear clusters in the final configuration have different compositions: one is CeNd_2_Sm_2_Tb (single unit cell *E*_mix_ = −6.6 kJ/mol and *E*_hull_ = 1.1 kJ/mol) and CeNd_2_TbEr_2_ (single unit cell *E*_mix_ = −12.3 kJ/mol and *E*_hull_ = 0.0 kJ/mol). Thus, the initial 5-metal configuration decomposes into two different and more stable 4-metal subclusters.

**Figure 8:**
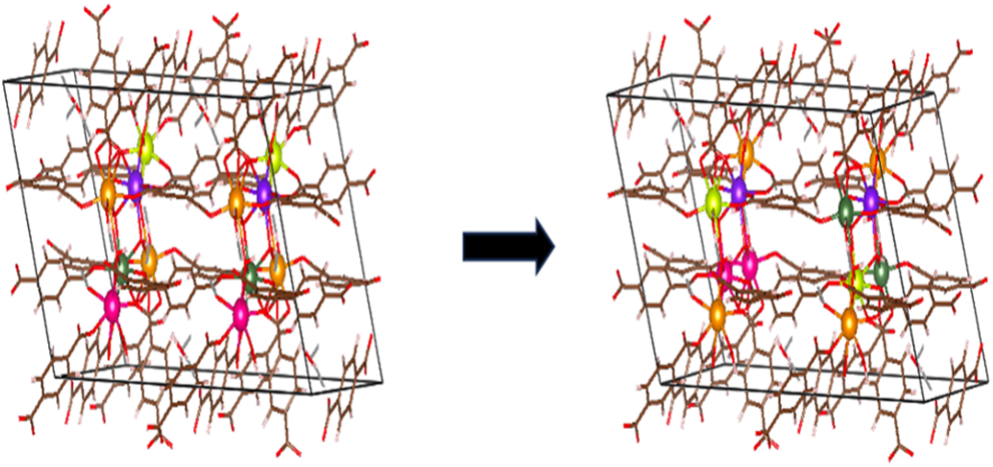
2 × 1 × 1 Supercell structure before and after performing the metal swapping algorithm for a metastable configuration of CeNd_2_SmTbEr. The color codes for all of the different metal atoms are as follows: Ce (neon green), Nd (orange), Sm (dark pink), Tb (purple), and Er (dark green).

We also performed similar calculations for three other metastable structures from the Ce–Nd–Tb–Sm−Er composition space, one structure each from 4-metal, 3-metal, and 2-metal MOF. In Figure S10, we show how these structures and their respective *E*_mix_ values change after 10 iterations. We observed similar metal swapping or clustering trends in the final configurations that result in significantly lower *E*_mix_ values.

In another scenario, we examined the influence of these structural aspects on several 2 × 1 × 1 supercells of structures that were identified as potentially stable in our previous convex hull calculations using single unit cells. Specifically, we selected one structure each from the 5-metal and 4-metal MOFs from the Ce–Nd–Tb–Sm−Er composition space. In Figure S11­(a,b), we show how these structures and their respective *E*_mix_ values change after 10 iterations. In each case, the lowest energy structure included a single metal swap relative to the initial configuration, with a change in the energy of mixing of less than or equal to 0.1 kJ mol^–1^·unit cell^–1^. We also applied this approach to 2-metal and 3-metal MOF 2 × 1 × 1 supercells of structures that lie on the convex hull (see Figure S11­(c,d)) and found similar observations.

These calculations with representative structures from our initial estimate of the convex hull suggest that our calculations with single unit cells are sufficient to estimate the overall convex hull of the multicomponent composition space. This is plausible for this prototype MOF because the physical separation of the hexanuclear clusters is likely to mean that contributions to the energy of mixing from cluster–cluster interactions (e.g., local strain in linkers connecting clusters) are smaller than the energies associated directly with bonds forming the cluster.

## Summary

Current research toward creating multifunctional HE-MOFs is still in its early stage. One of the primary challenges associated with these materials is the vast compositional landscape, which makes it difficult to rely on experimental trial-and-error methods for their synthesis. Additionally, factors such as local order and disorder at the unit cell level are difficult to directly probe experimentally. This situation suggests that leveraging high-throughput computational methods can help us to better understand the complex characteristics governing the stability of these materials.

We have used DFT as well as MLIP calculations to garner insights into the compositional landscape of high-entropy derivatives of a prototypical lanthanide-based MOF. While the parent crystal structure of the MOF is homometallic, we investigated derivative unit cells containing up to five different lanthanide species. Thermodynamically stable structures were identified by comparing energy of mixing values and performing convex hull calculations. Although these calculations cannot provide information about kinetic effects that may occur during the crystallization of real MOFs, our results indicate that only a small fraction of the many structures with multimetal compositions that exist lie on the convex hull. This situation points to the complexity associated with selecting predetermined compositions to validate synthetically viable HE-MOF candidates.

We also explored the effect of metal ordering on the energetic stability of these materials by examining several example supercell structures. This approach was found to be useful in finding more stable variants of structures that are metastable on the single unit cell level, but the changes in the energy of mixing for structures close to the convex hull were small.

Our analysis is limited to a specific prototype MOF, and our findings provide insights about the expected trends and complexities rather than making precise quantitative predictions about this material, as well as other HEMs. Because of the large number of compositional variants that are possible, we relied on MLIP-based calculations to systematically explore a wide range of structures. We showed that the specific MLIP we used gives results in reasonable agreement with DFT calculations, but that the energy variations between possible compositions can be small. We also noted that the MLIP we used (CHGNet) was suitable for only a subset of lanthanide metals. If calculations were to be used to make predictions about specific compositions of an MOF of interest in experimental studies, it would be wise to use additional DFT calculations generated by the MLIP-generated convex hull to refine the precision of the overall predictions.

## Supplementary Material





## Notes

Comparison between the DFT and MLIPs relative energies of the relaxed homometallic Ln-MOFs; compositions and the CHGNet *E*_mix_ of the structures lying on the convex hull for the two 5-metal MOF systems; detailed distribution of unique stable MOF structures lying on convex hulls; 2D regular pentagon projection of composition spaces; *E*_hull_ histograms; parity plots *E*_mix_ for CHGNet compared to DFT; 2 × 1 × 1 supercell structures before and after performing the metal swapping algorithm; INCAR file parameters for DFT calculations (PDF)
Tabulated data for figures (XLSX)
