# Supplementary material for: Computational Analysis of the Energetic Stability of High-Entropy Structures of a Prototypical Lanthanide-Based Metal–Organic Framework
Source: J Phys Chem C Nanomater Interfaces. 2025 Oct 9;129(42):19124–33. doi: 10.1021/acs.jpcc.5c04892 (PMC12557373; doi:10.1021/acs.jpcc.5c04892)
Supplement: Supplementary file 1 [file jp5c04892_si_001.pdf]

Supporting Information

Computational analysis of the energetic stability of high-entropy structures of a prototypical lanthanide-based metal-organic framework

Surbhi K. A. Kumar,[1] Dorina F. Sava Gallis,[2] and David S. Sholl[3*]

[1]*School of Chemical Biomolecular Engineering, Georgia Institute of Technology, Atlanta, Georgia 30332-0100, USA*

[2]*Nanoscale Sciences Department, Sandia National Laboratories, Albuquerque, New Mexico 87185-1415, USA*

[3] *University of Tennessee-Oak Ridge Innovation Institute, Oak Ridge National Laboratory, Oak Ridge, Tennessee 37831-6173,USA*

E-mail: shollds@ornl.gov

In addition to the Supporting Information below, dataset comprising of structure POSCAR files and python codes for the analysis described in the manuscript are available via GitHub at https://github.com/fvnper/HE-MOFs. An excel sheet consisting of the tabulated data from the figures in the main manuscript is also provided.

Supplementary Tables

Table S1: Comparison between the DFT and MLIPs relative energies of the relaxed homometallic Ln-MOFs. Cases in which the observed trends with DFT and the two MLIPs are very different are highlighted in bold.

Ln-MOF	DFT<br>$E_{Ln6} - E_{Tb6}$ (eV/unit cell)	M3GNet<br>$E_{Ln6} - E_{Tb6}$ (eV/unit cell)	CHGNet<br>$E_{Ln6} - E_{Tb6}$ (eV/unit cell)
La_6	-1.11	-1.68	-1.28
Ce_6	2.01	-4.71	-4.56
Pr_6	1.50	1.18	2.00
Nd_6	1.01	0.66	1.46
Pm_6	0.73	1.19	1.25
Sm_6	0.69	0.28	0.82
**Eu_6**	**0.51**	**-21.04**	**-22.79**
**Gd_6**	**-0.19**	**-55.26**	**-55.08**
Tb_6	0.00	0.00	0.00
Dy_6	-0.15	0.36	-0.05
Ho_6	0.04	0.36	0.14
Er_6	0.11	0.28	-0.05
Tm_6	-0.01	0.81	0.43
**Yb_6**	**-0.08**	**33.45**	**30.88**
Lu_6	0.03	0.51	0.53

Table S2: Compositions and the CHGNet E_{mix} (without ideal entropy) of the 97 structures lying on the convex hull for Ce-Nd-Sm-Er-Tb MOFs.

Ln-MOF	CHGNet E_{mix} (kJ.mol^{-1}.unit cell^{-1})
$Ce_2NdSmTbEr$	-8.8
$CeNd_2SmTbEr$	-11.1
$CeNd_2TbEr_2$	-12.3
$Ce_2NdSmEr_2$	-10.0
Ce_2Sm_2TbEr	-7.5
$Ce_2NdTbEr_2$	-9.7
$NdSm_3TbEr$	-8.1
Ce_2SmTb_2Er	-8.1
$CeNd_2SmTb_2$	-9.9
$Ce_3NdSmEr$	-6.5
$CeNdSm_2Er_2$	-11.0
$CeNd_3TbEr$	-9.9
$Nd_2SmTbEr_2$	-12.5
$CeNdSm_3Er$	-7.8
$NdSmTb_2Er_2$	-11.3
$CeSmTb_2Er_2$	-9.6
$CeNd_2SmEr_2$	-12.2
$CeNdSm_2Tb_2$	-8.4
$CeSmTbEr_3$	-9.4
$CeNdTb_2Er_2$	-11.6
$CeNdTbEr_3$	-11.1
Nd_3Sm_2Tb	-6.8
$CeNd_3Sm_2$	-5.3
Ce_4SmEr	-3.8
$CeTbEr_4$	-6.9
$CeSm_3Er_2$	-7.4
Nd_3TbEr_2	-11.7
Ce_2Nd_3Sm	-3.9
$NdSm_3Er_2$	-9.6
Ce_2NdSm_3	-4.1
$CeNd_2Er_3$	-11.1
Nd_4TbEr	-8.3
Ce_2Nd_3Tb	-4.8
$CeTb_3Er_2$	-6.5

Continued.

Nd_3SmEr_2	-11.0
$CeNd_3Er_2$	-10.7
Nd_3SmTb_2	-8.5
$NdSmEr_4$	-8.1
Nd_2TbEr_3	-12.9
$Nd_2Sm_2Tb_2$	-8.8
$Ce_2Tb_2Er_2$	-8.8
$Ce_2Nd_2Sm_2$	-5.4
Ce_3Sm_2Tb	-4.6
Ce_3TbEr_2	-7.0
Nd_3Tb_2Er	-10.4
$Sm_2Tb_2Er_2$	-9.4
$CeNdTb_4$	-8.1
$CeNd_2Tb_3$	-9.3
Nd_2Sm_3Tb	-6.9
$CeNdEr_4$	-10.6
Ce_3Nd_2Tb	-4.5
$CeNdSm_4$	-3.9
$SmTb_3Er_2$	-6.7
$CeNd_2Sm_3$	-5.6
Ce_3Tb_2Er	-6.7
$Ce_2Sm_2Er_2$	-7.3
Nd_2Sm_3Er	-8.9
$CeSmTb_4$	-6.1
$Ce_2Nd_2Tb_2$	-7.4
$Nd_2Tb_2Er_2$	-13.2
Ce_2Sm_3Tb	-4.8
Ce_3NdSm_2	-3.6
$CeSmEr_4$	-9.1
Sm_3Tb_3	-4.5
Nd_4Tb_2	-7.3
Nd_4Er_2	-8.3
Ce_2Nd_4	-2.3
Tb_4Er_2	-4.0
Ce_4Sm_2	-1.4
Tb_2Er_4	-5.8
Nd_2Er_4	-12.6
Nd_5Sm	-2.0
$CeTb_5$	-3.2
Nd_3Sm_3	-4.4

Continued.

Continued.

Nd_2Tb_4	-9.9
Ce_3Tb_3	-5.6
$NdSm_5$	-2.5
$CeSm_5$	-1.1
Tb_3Er_3	-4.9
Nd_5Er	-4.8
Nd_5Tb	-4.1
$CeNd_5$	-1.8
Sm_4Er_2	-5.8
Nd_4Sm_2	-4.0
$TbEr_5$	-3.2
Sm_2Er_4	-9.8
Nd_2Sm_4	-4.8
Sm_2Tb_4	-5.9
Ce_2Er_4	-7.8
Ce_5Nd	-0.7
Ce_3Sm_3	-2.0
Ce_2Tb_4	-5.5
Ce_6	0.0
Nd_6	0.0
Sm_6	0.0
Tb_6	0.0
Er_6	0.0

Table S3: Compositions and the CHGNet E_{mix} (without ideal entropy) of the 113 structures lying on the convex hull for Pr-Nd-Sm-Er-Tb MOFs.

Ln-MOF	CHGNet E_{mix} (kJ.mol^{-1}.unit cell^{-1})
Pr_2NdSm_2Tb	-9.4
$PrSm_3TbEr$	-9.4
$PrNd_2Sm_2Tb$	-8.2
$PrNd_2Tb_2Er$	-11.5
$Pr_2NdSmEr_2$	-13.3
$PrNdSm_3Er$	-10.2
Pr_2Sm_2TbEr	-13.1
$PrNdTb_2Er_2$	-13.7
Pr_2NdTb_2Er	-12.4
$Nd_2SmTbEr_2$	-12.5
$PrNdSm_3Tb$	-9.0
$NdSm_3TbEr$	-8.1
Pr_2Nd_2SmTb	-8.4
$NdSmTb_2Er_2$	-11.3
$Pr_3NdSmTb$	-7.9
Pr_2Nd_2TbEr	-10.9
$PrNdTb_3Er$	-12.3
$Pr_2NdTbEr_2$	-13.7
$Pr_2NdSmTb_2$	-11.1
$PrNdSm_2Tb_2$	-11.0
$PrSmTb_2Er_2$	-11.8
Pr_2SmTb_2Er	-13.6
$PrNdSm_2Er_2$	-13.3
Pr_3NdTb_2	-9.4
$PrNd_4Tb$	-5.6
Pr_3TbEr_2	-12.8
Pr_3NdEr_2	-11.6
Nd_3TbEr_2	-11.7
Pr_2Nd_3Tb	-7.0
$Pr_2Nd_2Sm_2$	-6.5
$Pr_2Sm_2Er_2$	-14.3
Pr_4NdTb	-5.9
$Sm_2Tb_2Er_2$	-9.4
$PrNdEr_4$	-12.7
Nd_3Tb_2Er	-10.4

Continued.

Pr_2NdTb_3	-11.1
$PrNd_3Er_2$	-10.8
Nd_2Sm_3Tb	-6.9
$NdSm_3Er_2$	-9.6
Pr_2Tb_3Er	-12.8
$PrNd_2Tb_3$	-10.0
Pr_3Sm_2Tb	-9.0
Pr_3Sm_2Er	-9.9
Pr_2Nd_3Er	-7.6
$PrNdSm_4$	-6.2
Nd_4TbEr	-8.3
$PrSmTb_4$	-8.5
$Nd_2Sm_2Tb_2$	-8.8
$PrNdTb_4$	-10.6
Nd_3SmTb_2	-8.5
$NdSmTb_4$	-8.1
Nd_3SmEr_2	-11.0
Pr_2Sm_3Er	-11.2
$PrNd_4Er$	-6.4
Pr_3Nd_2Tb	-6.6
$PrNd_3Tb_2$	-8.7
Pr_2TbEr_3	-13.6
$Pr_2Nd_2Tb_2$	-9.8
Pr_3Tb_2Er	-11.8
$PrNd_2Sm_3$	-6.1
Pr_3NdSm_2	-6.2
Pr_4NdSm	-4.1
Pr_3SmTb_2	-10.5
Nd_2TbEr_3	-12.9
$Pr_2Tb_2Er_2$	-14.1
$Pr_2Nd_2Er_2$	-12.0
Pr_4SmTb	-7.2
Pr_3SmEr_2	-12.6
Pr_2Nd_3Sm	-5.3
Pr_2Sm_3Tb	-10.4
Nd_3Sm_2Tb	-6.8
$SmTb_3Er_2$	-6.7
Nd_2Sm_3Er	-8.9
Pr_2NdSm_3	-7.6
Pr_2SmTb_3	-12.2

Continued.

$Nd_2Tb_2Er_2$	-13.2
Nd_4Tb_2	-7.3
Nd_4Er_2	-8.3
Tb_4Er_2	-4.0
Pr_5Er	-5.6
Pr_3Sm_3	-7.1
$NdSm_5$	-2.5
Sm_3Tb_3	-4.5
Pr_2Tb_4	-11.0
Pr_4Tb_2	-8.9
Nd_3Sm_3	-4.4
Tb_2Er_4	-5.8
Nd_2Er_4	-12.6
Nd_5Sm	-2.0
Nd_2Tb_4	-9.9
Pr_4Er_2	-10.8
Pr_3Tb_3	-10.4
$PrNd_5$	-1.4
Nd_5Tb	-4.1
Pr_3Nd_3	-3.6
Nd_5Er	-4.8
Nd_2Sm_4	-4.8
Sm_4Er_2	-5.8
Pr_2Sm_4	-7.2
Sm_2Er_4	-9.8
$TbEr_5$	-3.2
Pr_5Nd	-1.3
Nd_4Sm_2	-4.0
Tb_3Er_3	-4.9
Pr_4Sm_2	-5.3
Pr_5Tb	-5.0
Sm_2Tb_4	-5.9
Pr_2Er_4	-12.7
Pr_6	0.0
Nd_6	0.0
Sm_6	0.0
Tb_6	0.0
Er_6	0.0

Supplementary Figures

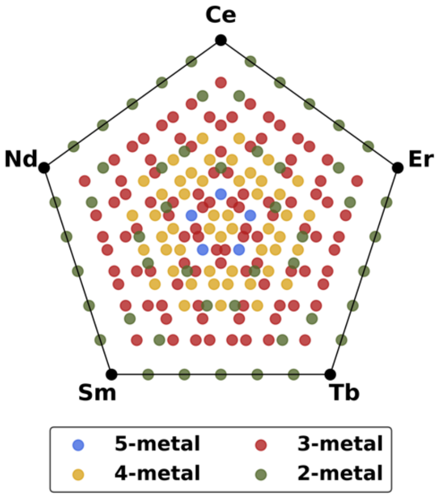

Figure S1: 2D regular pentagon coordinates projection of Ce-Nd-Sm-Tb-Er MOF composition space highlighting the composition of the MOF structures lying on the convex hull determined using CHGNet E_{mix} with ideal configurational entropy at 900 K.

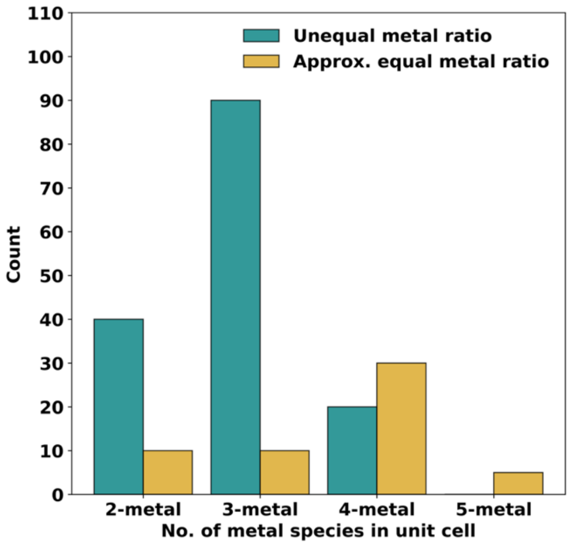

Figure S2: Distribution of unique stable MOF structures lying on the convex hull for Ce-Nd-Sm-Tb-Er MOFs determined using CHGNet E_{mix} with ideal configurational entropy at 900 K.

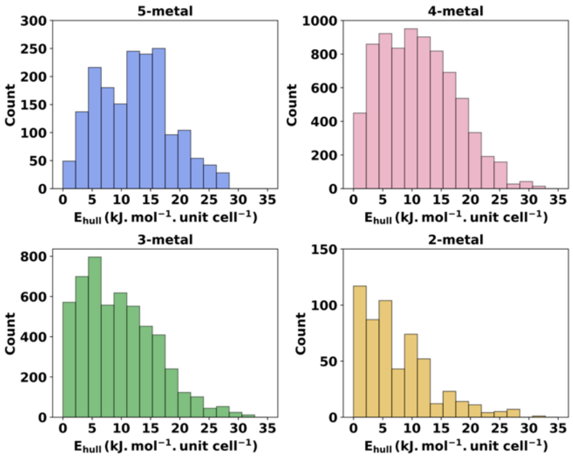

(a)

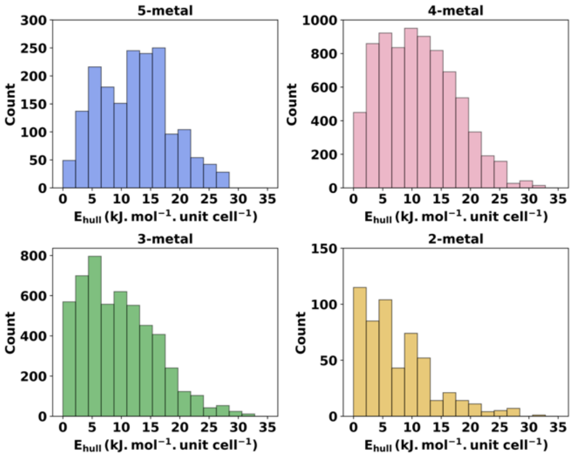

(b)

Figure S3: Histograms of E_{hull} values determined for all the metastable structures from the convex hull analysis for Ce-Nd-Sm-Tb-Er MOFs determined using CHGNet E_{mix} with ideal configurational entropy: (a) 300 K, and (b) 900 K.

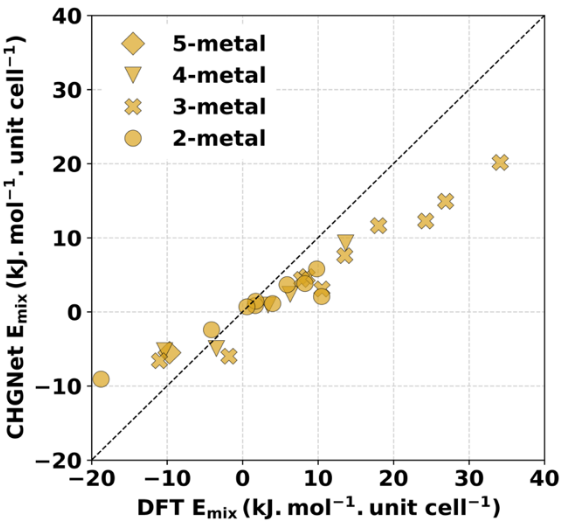

Figure S4: Parity plot of CHGNet and DFT E_{mix} for 26 different configurations of the prototype MOF after substituting Ce-based structures with Pr-based ones. The mean absolute error (MAE) is 5.0 $kJ.mol^{-1}.unit\ cell^{-1}$ (0.052 eV/unit cell).

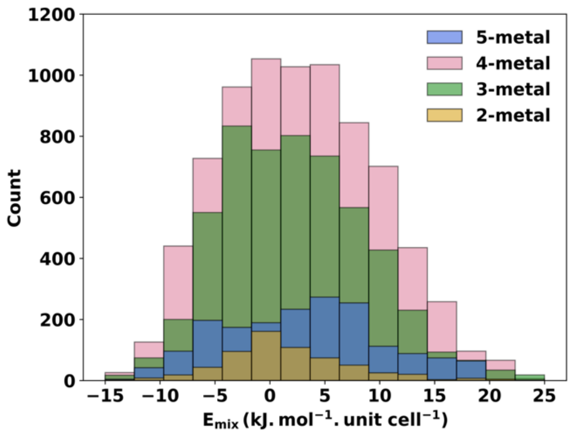

Figure S5: Histograms of CHGNet E_{mix} values for all the single unit cell structures of the prototype MOF within Pr-Nd-Sm-Tb-Er MOF composition space.

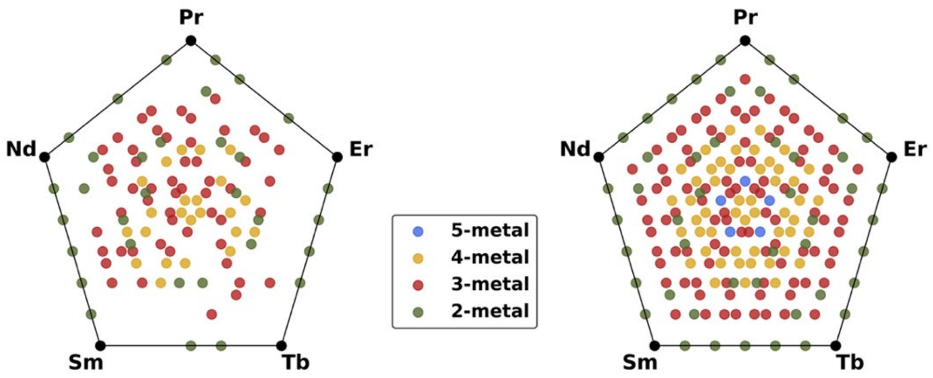

(a) (b)

Figure S6: 2D regular pentagon coordinates projection of Pr-Nd-Sm-Tb-Er MOF composition space highlighting the composition of the MOF structures lying on the convex hull determined using energies from CHGNet: (a) E_{mix} without inclusion of configurational entropy, and (b) E_{mix} with ideal configurational entropy at 300 K.

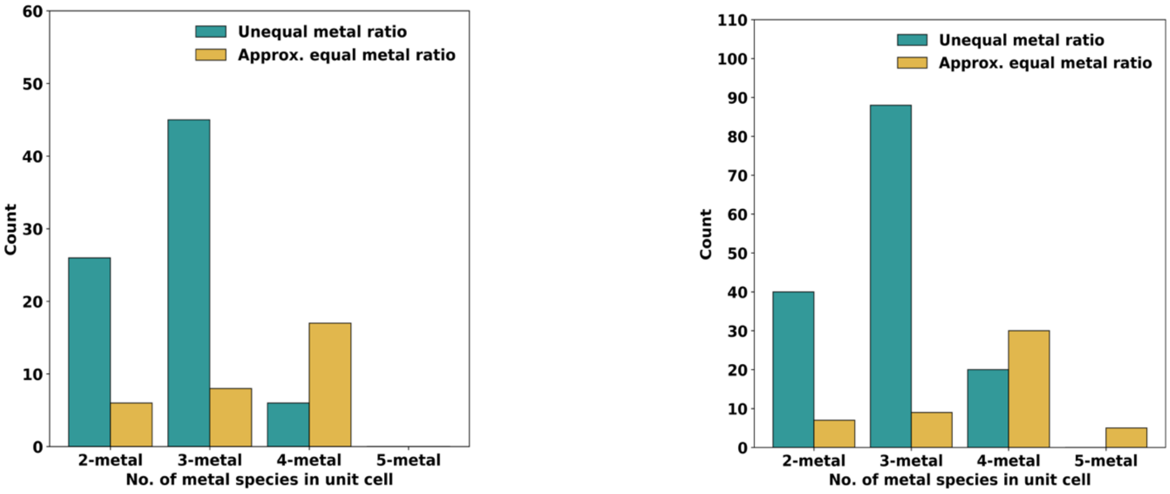

(a) (b)

Figure S7: Distribution of unique stable MOF single unit cell structures lying on the Pr-Nd-Sm-Tb-Er convex hull determined using energies from CHGNet: (a) E_{mix} without inclusion of configurational entropy, and (b) E_{mix} with ideal configurational entropy at 300 K.

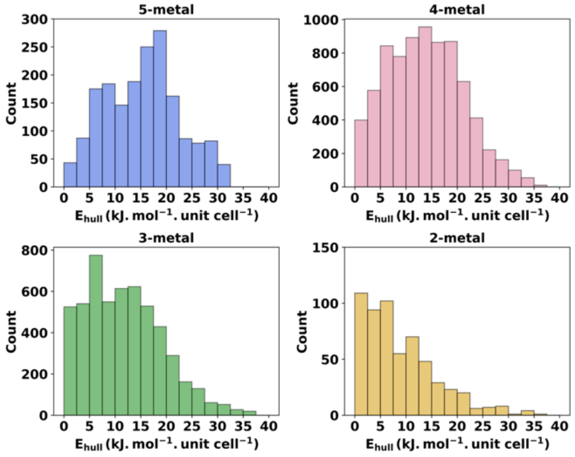

(a)

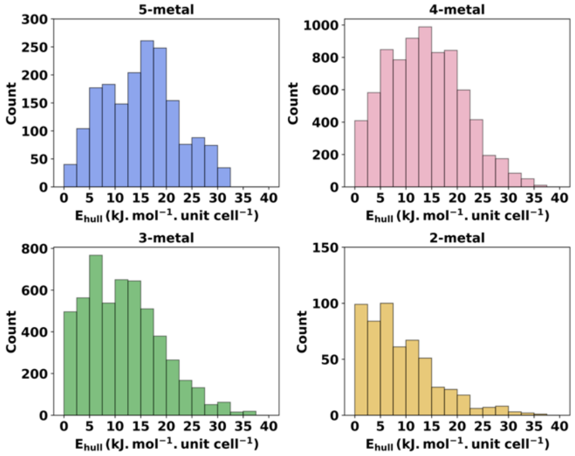

(b)

Figure S8: Histograms of E_{hull} values determined for all the metastable structures from the convex hull analysis for Pr-Nd-Sm-Tb-Er MOFs determined using energies from CHGNet: (a) E_{mix} without inclusion of configurational entropy, and (b) E_{mix} with ideal configurational entropy at 300 K.

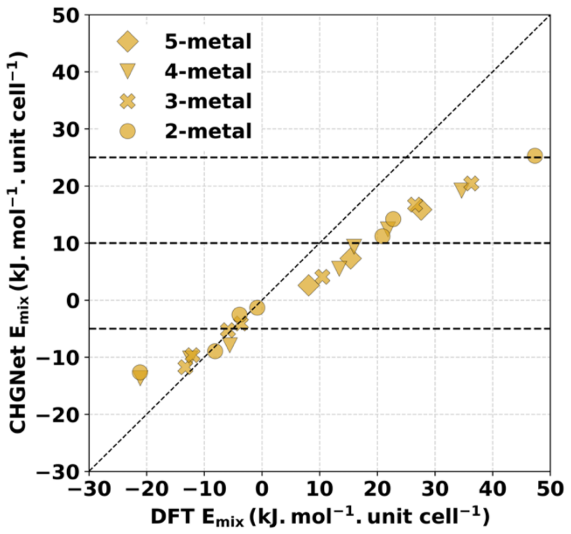

Figure S9: Parity plot of CHGNet and DFT E_{mix} for 24 heterometallic configurations of the prototype MOF within Pr-Nd-Sm-Tb-Er composition space. 11 of the structures with CHGNet E_{mix} < 0 lie on the convex hull determined with this force field. The MAE for structures lying on the convex hull is 2.6 kJ.mol^{-1}.unit cell^{-1}. Total MAE for the 24 structures is 6.9 kJ.mol^{-1}.unit cell^{-1}.

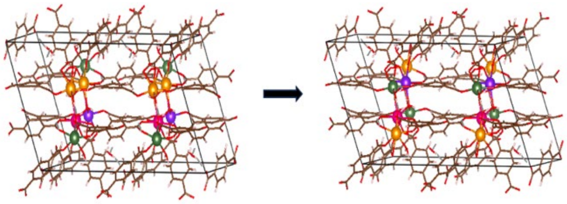

(a)

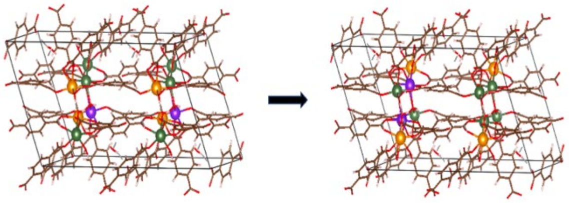

(b)

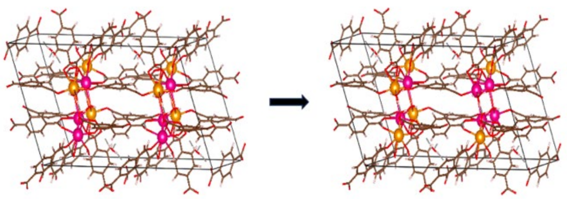

(c)

Figure S10: 2×1×1 supercell structure before and after performing the metal swapping algorithm for three metastable configurations from the Ce-Nd-Sm-Tb-Er convex hull analysis using energies from CHGNet: (a) 4-metal MOF ($Nd_2SmTbEr_2$) (Initial E_{mix} = 17.2 kJ.mol^{-1}.unit cell^{-1} and final E_{mix} = -9.6 kJ.mol^{-1}.unit cell^{-1}), (b) 3-metal MOF (Nd_2TbEr_3) (Initial E_{mix} = 15.3 kJ.mol^{-1}.unit cell^{-1} and final E_{mix} = -11 kJ.mol^{-1}.unit cell^{-1}), and (c) 2-metal MOF (Nd_3Sm_3) (Initial E_{mix} = -1.5 kJ.mol^{-1}.unit cell^{-1} and final E_{mix} = -4.2 kJ.mol^{-1}.unit cell^{-1}). The color codes for all the different metal atoms are as follows: Nd (orange), Sm (dark pink), Tb (purple), and Er (dark green).

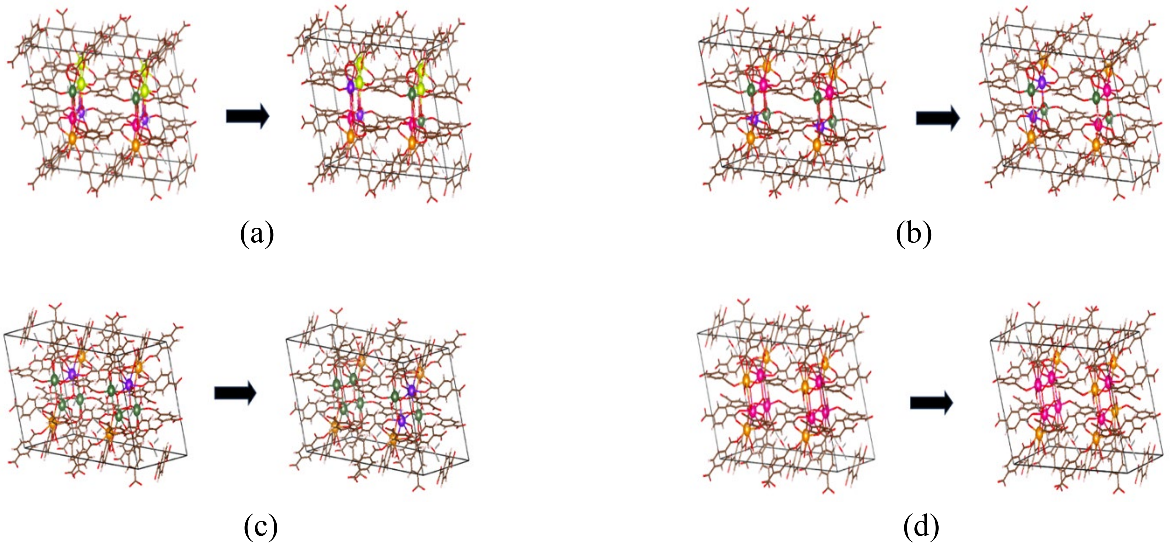

Figure S11: 2×1×1 supercell structure before and after performing the metal swapping algorithm for four stable configurations lying on the Ce-Nd-Sm-Tb-Er convex hull determined using energies from CHGNet: (a) 5-metal MOF ($Ce_2NdSmTbEr$) (Initial E_{mix} = -8.9 kJ.mol^{-1}.unit cell^{-1} and final E_{mix} = -8.9 kJ.mol^{-1}.unit cell^{-1}), (b) 4-metal MOF ($Nd_2SmTbEr_2$) (Initial E_{mix} = -12.5 kJ.mol^{-1}.unit cell^{-1} and final E_{mix} = -12.6 kJ.mol^{-1}.unit cell^{-1}), (c) 3-metal MOF (Nd_2TbEr_3) (Initial E_{mix} = -12.9 kJ.mol^{-1}.unit cell^{-1} and final E_{mix} = -9.6 kJ.mol^{-1}.unit cell^{-1}), and (d) 2-metal MOF (Nd_3Sm_3) (Initial E_{mix} = -4.5 kJ.mol^{-1}.unit cell^{-1} and final E_{mix} = -4.5 kJ.mol^{-1}.unit cell^{-1}). The color codes for all the different metal atoms are as follows: Ce (neon green), Nd (orange), Sm (dark pink), Tb (purple), and Er (dark green).

DFT optimization: VASP INCAR file parameters

IBRION = 2
GGA = PS
IVDW = 12
ISIF = 2 (set to 3 in case of volume relaxation)
ENCUT = 520
MAXMIX = 50
NELM = 400
EDIFF = 1E-05
EDIFFG = -0.01
ISMEAR = 0
SIGMA = 0.01
NSW = 1000
POTIM = 0.01
LREAL = AUTO
LWAVE = .TRUE.
LCHARG = .TRUE.
LMAXMIX = 6

VASP SPE INCAR file parameters

GGA = PS
IVDW = 12
ENCUT = 520
MAXMIX = 50
NELM = 400
EDIFF = 1E-05
EDIFFG = -0.01
ISMEAR = 0
SIGMA = 0.01
LREAL = AUTO
LWAVE = .TRUE.
LCHARG = .TRUE.
LMAXMIX = 6
